# GABA Concentrations in the Anterior Cingulate Cortex Are Associated with Fear Network Function and Fear Recovery in Humans

**DOI:** 10.3389/fnhum.2017.00202

**Published:** 2017-04-27

**Authors:** Nina Levar, Judith M. C. van Leeuwen, Nicolaas A. J. Puts, Damiaan Denys, Guido A. van Wingen

**Affiliations:** ^1^Department of Psychiatry, Academic Medical CenterAmsterdam, Netherlands; ^2^Brain Imaging Center, Academic Medical CenterAmsterdam, Netherlands; ^3^Amsterdam Brain and Cognition, University of AmsterdamAmsterdam, Netherlands; ^4^Spinoza Center for NeuroimagingAmsterdam, Netherlands; ^5^Department of Psychiatry, University Medical Center UtrechtUtrecht, Netherlands; ^6^Russell H. Morgan Department of Radiology and Radiological Science, Johns Hopkins UniversityBaltimore, MD, USA; ^7^FM Kirby Center for Functional Brain Imaging, Kennedy Krieger InstituteBaltimore, MD, USA

**Keywords:** fear, extinction, recovery, fMRI, MRS, GABA, SCR, dACC

## Abstract

Relapse of fear after successful treatment is a common phenomenon in patients with anxiety disorders. Animal research suggests that the inhibitory neurotransmitter γ-aminobutyric acid (GABA) plays a key role in the maintenance of extinguished fear. Here, we combined magnetic resonance spectroscopy and functional magnetic resonance imaging to investigate the role of GABA in fear recovery in 70 healthy male participants. We associated baseline GABA levels in the dorsal anterior cingulate cortex (dACC) to indices of fear recovery as defined by changes in skin conductance responses (SCRs), blood oxygen level dependent responses, and functional connectivity from fear extinction to fear retrieval. The results showed that high GABA levels were associated with increased SCRs, enhanced activation of the right amygdala, and reduced amygdala-ventromedial prefrontal cortex connectivity during fear recovery. Follow-up analyses exclusively for the extinction phase showed that high GABA levels were associated with reduced amygdala activation and enhanced amygdala-ventromedial prefrontal cortex connectivity, despite the absence of correlations between GABA and physiological responses. Follow-up analyses for the retrieval phase did not show any significant associations with GABA. Together, the association between GABA and increases in SCRs from extinction to retrieval, without associations during both phases separately, suggests that dACC GABA primarily inhibits the consolidation of fear extinction. In addition, the opposite effects of GABA on amygdala activity and connectivity during fear extinction compared to fear recovery suggest that dACC GABA may initially facilitate extinction learning.

## Introduction

The experience of a dangerous or harmful situation often results in the implicit association of a neutral stimulus with the fear experience. This stimulus can then elicit a fear response, even in the absence of the potentially aversive outcome, which is seen in patients with anxiety disorders. Exposure therapy makes use of extinction processes and is aimed at overcoming these inappropriate responses by repeatedly exposing individuals to the feared stimulus, gradually resulting in a new safe association which inhibits the old fear memory (Bouton, 2004). Despite short-term benefits of exposure therapy, relapse into fear is a common problem in the treatment of anxiety disorders such as post-traumatic stress disorder (PTSD) (Durham et al., 2005; Milad et al., 2008; Duits et al., 2015). This phenomenon is known as the spontaneous recovery of fear and is highly variable between individuals (Pavlov, 1927; Milad and Quirk, 2002; Bush et al., 2007).

Theoretical accounts posit that spontaneous recovery is the consequence of reduced inhibition of conditioned responses (Rescorla, 2004), and pharmacological studies in rodents point to a key role of the primary inhibitory neurotransmitter γ-aminobutyric acid (GABA) in the acquisition and maintenance of fear inhibition. These studies indicate that increased GABAergic neurotransmission during extinction training initially reduces fear expression. In contrast, increasing GABAergic neurotransmission disrupts the consolidation of the acquired extinction memory which consequently leads to stronger recovery of fear (Bindra et al., 1965; Bouton et al., 1990; McGaugh et al., 1990; Makkar et al., 2010).

Despite growing insight into the neurocircuitry of fear related processes, the underlying neurochemical basis of human fear learning is largely unknown. While rodent studies have investigated manipulations of GABAergic neurotransmission using high pharmacological doses, little is known about the role of natural variations in endogenous GABA levels. Proton magnetic resonance spectroscopy (^1^H-MRS) enables us to non-invasively assess brain metabolite concentrations *in vivo* and to relate these to individual differences in BOLD responses and cognitive processes (Northoff et al., 2007; Muthukumaraswamy et al., 2009; Sumner et al., 2010). A recent study pointed to a dysregulation of prefrontal GABA in anxiety disorders by showing that GABA levels in the medial prefrontal cortex (mPFC) in PTSD patients are increased (Michels et al., 2014). In line with this, Delli Pizzi et al. (2016b) showed that GABA levels in the vmPFC were positively correlated with anxiety as scored by the State Trait Anxiety Inventory (STAI-Y2) in healthy individuals.

In humans, the dorsal anterior cingulate cortex (dACC) as part of the mPFC has been shown to play a key role in the reappraisal of negative emotions and the expression of fear during fear conditioning (Milad et al., 2007; Sehlmeyer et al., 2009; Etkin et al., 2011). The dACC, ventromedial (vmPFC), and amygdala are tightly interconnected and compose a core circuit within a comprehensive fear network (Phelps et al., 2004; Ghashghaei et al., 2007; Milad et al., 2007; Shin and Liberzon, 2010; Klavir et al., 2013). Amygdala reactivity has been shown to covary with individual differences in fear responses and conditionability, rather than being representing fear responses in general (Bach et al., 2011; MacNamara et al., 2015). The vmPFC is primarily linked to extinction learning and the later retrieval of extinction and has been indicated to play a role in extinction learning with a proposed regulatory function of fear responses by exerting a modulatory impact on the amygdala (Quirk et al., 2003; Rosenkranz et al., 2003; Motzkin et al., 2015). The interplay between the amygdala and both mPFC and dACC has been indicated in memory consolidation following a fear conditioning and extinction paradigm (Feng et al., 2014). Resting fMRI revealed enhanced amygdala-dACC coupling, and decreased amygdala-mPFC coupling during memory consolidation following fear learning and extinction. Furthermore, a recent study showed that resting activity in the amygdala and the vmPFC were anti-correlated, with both trait anxiety scores and mPFC GABA levels being inversely correlated with amygdala-vmPFC coupling (Delli Pizzi et al., 2016a).

In the present study, we investigated associations of individual GABA concentrations in the dACC of healthy male volunteers with the recovery of fear, and assessed how GABA levels contribute to the consolidation and retrieval of previously extinguished fear. We focused on the influence of GABA on three core structures of the fear network: the amygdala, the vmPFC, and the dACC. On the basis of the above outlined work in rodents, we hypothesized that high GABA concentrations would initially facilitate extinction learning while subsequently hamper the consolidation of extinction learning, which is expected to result in heightened recovery of previously extinguished fear.

## Materials and methods

### Participants

**Figure 1:**
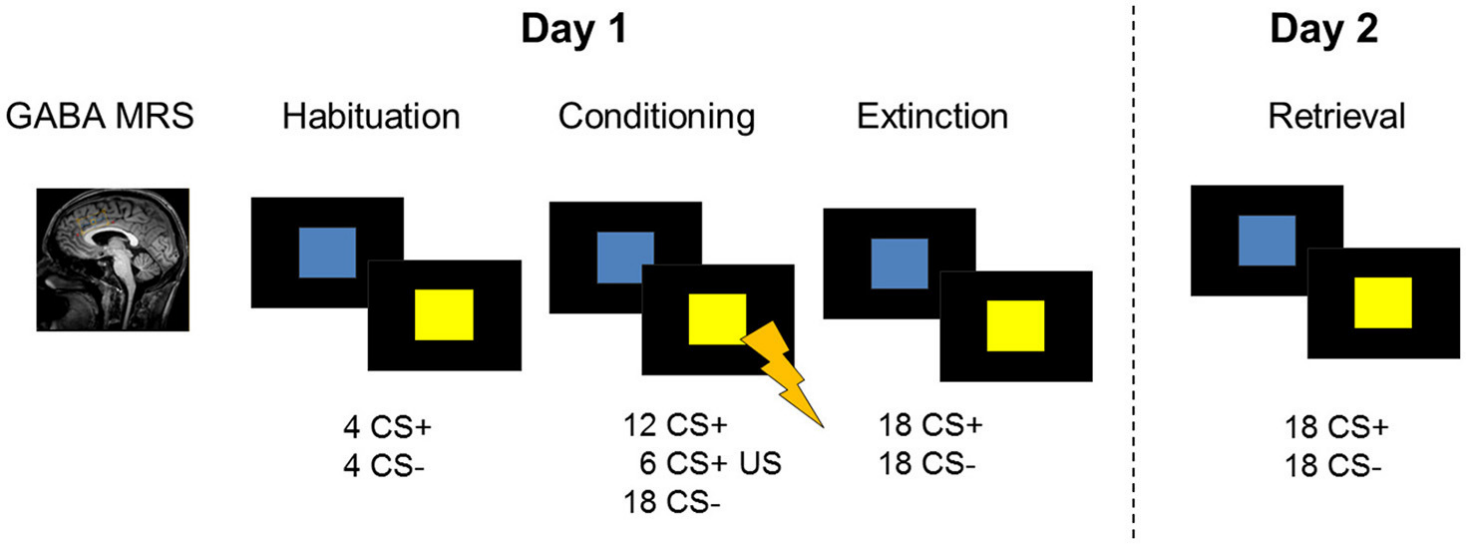
**Description of the 2-day study design including GABA MRS of the dACC and a fear conditioning and extinction paradigm during fMRI on experimental day 1**. On the following day, participants were tested for memory retrieval during fMRI. Skin conductance responses were recorded as a psychophysiological measure of on both days.

To investigate the impact of GABA on the recovery of fear, 106 healthy right-handed men between the ages of 18–35 years (*M* = 21.75 years, *SD* = 3.17) participated in a 2-day multi-modal MRI study (Figure 1; Supplemental Information for details). Data were unavailable or excluded due to one or more of the following reasons: technical problems (nine missing data sets), poor quality of the MRS spectra (27; see below), and skin conductance response (SCR) related technical issues during recordings (day 1: fifteen; day 2: seventeen). As extinction learning and extinction memory retrieval is dependent on successful conditioning procedures, six participants were excluded from the analysis as they showed an average SCR in the last four trials of the conditioning phase below 2 standard deviations of the group mean. Accordingly, data of 70 subjects were complete for inclusion in fMRI-MRS correlation analyses, and data of 57 subjects were complete for inclusion in the SCR-MRS correlation analyses. Informed consent was obtained before entering the study. The study was performed in accordance with the declaration of Helsinki and approved by the Medical Ethics Committee of the Academic Medical Center in Amsterdam.

### Procedures

Participants completed a 2-day fear conditioning protocol in a 3T whole-body MRI scanner (Philips, 3.0T Achieva) equipped with a 32-channel SENSE head-coil. On day one, a ^1^H MEGA-PRESS (Mescher et al., 1998; Waddell et al., 2007) scan of the dACC was acquired before undergoing a fear conditioning and extinction session during functional MRI (for details on acquisition parameters, see Supplemental Information). Approximately 24 h later, participants returned to test extinction retrieval during functional MRI. During the conditioning paradigm, mild electrical stimulation to the participants' right wrist was used as unconditioned stimulus. Individual stimulation intensity settings were determined prior to the experimental session by ramping up the current in small steps (2 mA) until the participant described the sensation as “highly annoying but not painful.” Skin conductance responses were recorded as a psychophysiological measure of fear throughout the functional MRI sessions.

### Fear conditioning and extinction task

The fear conditioning task included two conditioned stimuli (CSs) which consisted of a blue and a yellow square. On the first experimental day, the paradigm started with a habituation phase (8 trials) and was followed by the conditioning phase (36 trials) during which one stimulus (CS+) was paired with an unconditioned aversive stimulus (US) in 33% of the trials. The unconditioned stimulus consisted of a mild electric shock to the participant's right wrist and was administered at stimulus offset. US intensities were determined before the start of the scan in order to ascertain that the electric stimulus was unpleasant but not painful (see Supplemental Information). The other stimulus (CS−) was never paired with the US. The color of the CS+ was counterbalanced across subjects. Stimuli were presented for 4 s with an inter-stimulus interval ranging between 6.5 and 9.5 s. Shortly (20 s) after the conditioning phase, a fear extinction training was performed. During the extinction phase (36 trials), all conditioned stimuli were shown in the absence of the US. Instructions were presented on screen before every task phase, explaining that colored squares will be presented on screen and that shocks may be administered during the task. Further instructions stated that there is a relationship between the squares and the shock. On the second experimental day, SCR recordings were prepared as on day 1 and stimulation intensities were determined again. Extinction retrieval was tested by presenting the CSs in the absence of the unconditioned stimulus during fMRI (24 trials).

### Skin conductance responses

Skin conductance data were analyzed using NetStation software (Electrical Geodesics, Inc., Oregon, United States). A NetStation filter was used to remove large artifacts resulting from MR signals using a 2 TR window. Continuous SCR data was further analyzed using Matlab (Matlab Release 2014a, The MathWorks, Inc., Natick, Massachusetts, United States) and EEGLAB for Matlab (Delorme and Makeig, 2004). A median filter and a low pass filter were applied to remove remaining MR artifacts. SCR changes in response to stimulus presentation were quantified using the minimum response between stimulus onset and 2 s post-stimulus onset to accommodate the ongoing decrease in SCR to the previous stimulus. The maximum response between stimulus onset and 8 s post-stimulus was extracted to calculate the change in SCR to the stimulus. Due to large inter-individual differences in SCRs, data was standardized to the largest elicited SCRs (max(SCR)), which were the unconditioned responses to the shocks. We used the average of the SCRs (Avg_max(SCR)_) to the US responses to standardize data (SCR/Avg_max(SCR)_). In order to investigate the recovery of fear, differences in fear responses between the two experimental days were calculated. Including SCRs to both the extinguished stimuli on day 1 and responses to the retrieved stimulus on day 2, enabled us to take individual differences in fear extinction into account, as SCRs to the CSs varied between participants at the end of extinction learning. As there is no gold standard, a fear recovery index for skin conductance responses was defined for each participant as the differential response to the first trial during extinction retrieval (when fear recovery is maximal followed by rapid re-extinction) and the mean response to the last two trials during extinction training, to increase signal to noise by averaging over maximally extinguished trials. SPSS (IBM Corp. Released 2011. IBM SPSS Statistics for Windows, Version 20.0. Armonk, NY: IBM Corp) was used for statistical testing. Non-parametric tests (Wilcoxon–Mann–Whitney test) were used to assess differences in skin conductance responses due to CS types within and between phases. Non-parametric correlation analyses (Spearman's) were performed between GABA concentrations and SCRs of fear recovery, extinction retrieval, and extinction.

### MRS

**Figure 2:**
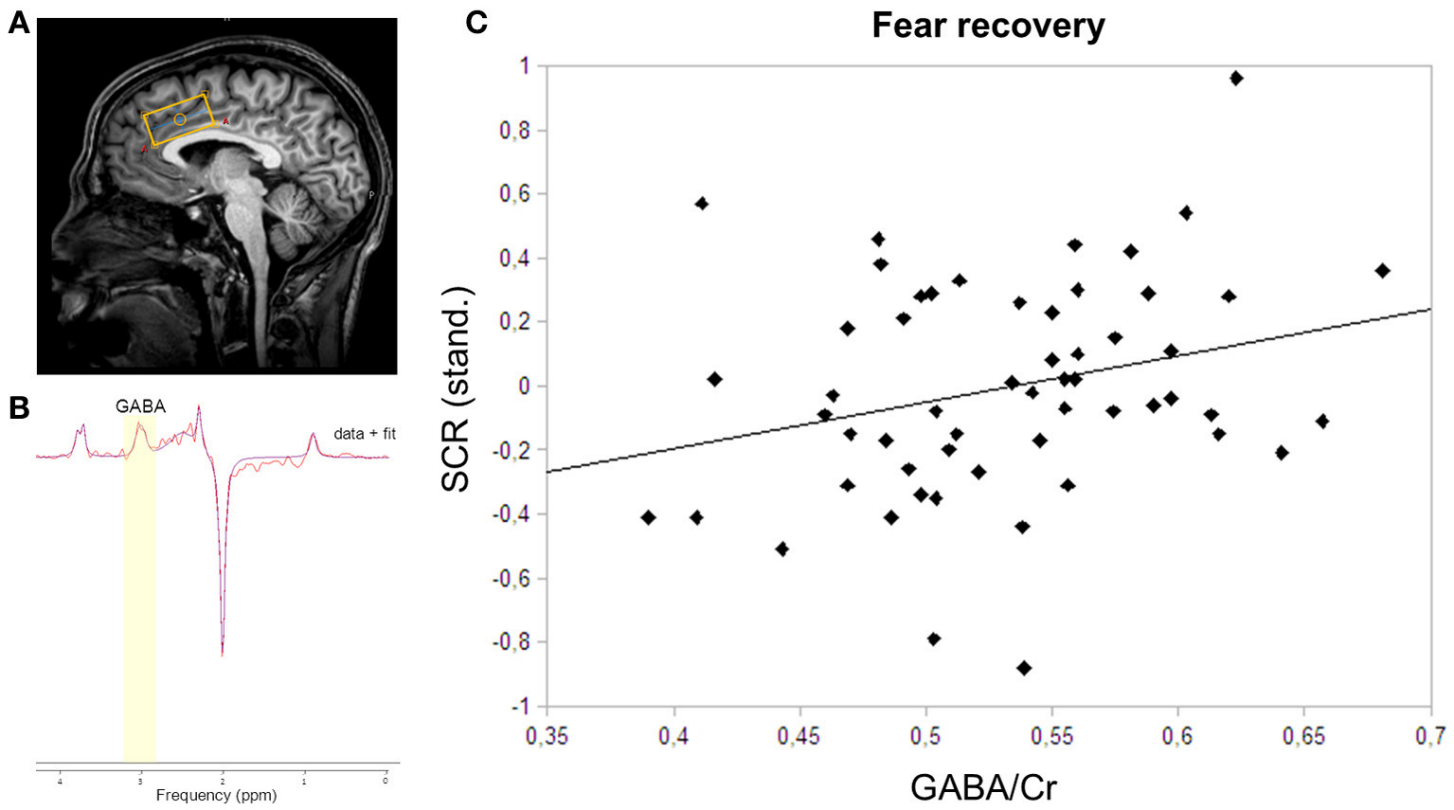
**MRS voxel placement, MRS spectrum with fit, and correlation plot of GABA and skin conductance responses. (A)** The MRS voxel was planned on the anatomical scan of each participant. The 40 × 20 × 20 mm^3^ box was closely aligned with the corpus callosum in the dACC. **(B)** GABA was fitted onto the spectrum using AMARES in jMRUI. GABA was fitted using a Gaussian function at 3.0 ppm. **(C)** The correlation of dACC GABA concentrations and skin conductance responses to the CS+ relative to the CS− during fear recovery revealed a positive relation (*r*_*s*_ = 0.28; *P* = 0.04).

GABA-edited MRS spectra were acquired from a 40 × 20 × 20 mm^3^ volume placed in the dorsal anterior cingulate cortex. The voxel was placed and tilted for the lower long side to be closely aligned with the corpus callosum (Figure 2A). GABA MRS spectra were acquired using a MEGA-PRESS sequence with the following parameters: TR/TE = 2,000/73, 384 averages, T_Acq_ = 12:48 min (Mescher et al., 1998; Waddell et al., 2007). Spectra were analyzed using AMARES in jMRUI, a non-linear –least-squares quantitation algorithm (Naressi et al., 2001). In a first step, any residual water signal was removed using the Hankel Lanczos singular value decomposition (HLSVD) filter. Pre-processing of both unedited and difference spectra included apodization (Lorentzian 5 Hz), zero filling (1,024), and zeroth-order phase correction with respect to NAA. Difference spectra were phased at 180°. GABA+ (GABA and macro-molecules) was fitted onto the difference spectra at 3.0 ppm using a single Gaussian function (Figure 2B). Unedited spectra were fitted for NAA (2.0 ppm), Cr+ (3.0 ppm), and Cho (3.2 ppm) using a Lorentzian curve. Creatine was used as an internal standard for GABA+ quantification. All spectra were independently rated by two of the researchers and 27 spectra were excluded from further analysis due to poor quality. Both difference and unedited spectra were excluded if the line width of the NAA peak exceeded 11 Hz. Importantly, the MRS quality control was performed independently from the subsequent analyses.

### MRI data analysis

Functional MRI data were analyzed using the Statistical Parametric Mapping toolbox (SPM8; Wellcome Trust Center for Neuroimaging, London, UK). Standard pre-processing steps were applied including realignment, slice time correction, co-registration to structural images, normalization into Montreal Neurological Image template space using the segmentation procedure, and spatial smoothing (8 mm FMWH). Data of the two experimental days were analyzed separately using a linear convolution model of the canonical hemodynamic response function. Low frequency drift was removed using a high pass filter (1/128 Hz). A design matrix which involved separate regressors for CS+ and CS− trials for each early (i.e., the first 6 CS+ and CS- trials) and late phases (i.e., the last six CS+ and CS− trials) of habituation, conditioning, and extinction for data of day one was defined for each participant. For the conditioning phase, reinforced and non-reinforced CS+ trials were modeled separately. For further analyses, only the non-reinforced of all CS+ trials were analyzed to avoid measuring BOLD responses to the US. Data of the second experimental session involved experimental conditions for early and late CS+ and CS− trials of the retrieval phase. The six parameters obtained from the realignment procedure were added as regressors of no interest into the design matrix. The obtained contrast images were then entered into a second level full-factorial design in order to assess task effects. A voxel-wise correlation analysis was performed to test for correlations between GABA concentrations and BOLD responses. GABA+/Cr+ ratios were added as covariates to the full-factorial design including CS+ and CS− responses during the late phase of extinction and the early phase of extinction retrieval. To assess the recovery of fear, the differential response (CS+ > CS) during the early phase of retrieval was compared to the differential response during the late phase of extinction (early retrieval_(*CS*+ > *CS*−)_> late extinction _(*CS*+ > *CS*−)_). To investigate functional coupling between brain regions, we used a generalized psychophysiological interaction analysis (gPPI) to assess connectivity of the right amygdala with all other brain voxels (http://brainmap.wisc.edu/PPI; McLaren et al., 2012). Generalized PPI analysis allows for the assessment of functional connectivity and interactions between different brain regions in a task-dependent manner by incorporating multiple contrasts. We included our contrasts of interest (i.e., retrieval and extinction: CS+ > CS−) and used GABA levels as covariates to examine correlations between dACC GABA and functional connectivity. The amygdala seed was defined as a 5 mm sphere around the group peak activity of GABA modulated fear recovery within the amygdala obtained from second level analysis.

All statistical tests were family-wise error (FWE) rate corrected for multiple comparisons across the whole brain on cluster level (*p* < 0.05) with an initial height threshold of *p* < 0.001 (Eklund et al., 2016), and statistical tests for the three regions of interest (dACC, amygdala, and vmPFC) were FWE rate corrected (*p* < 0.05) on voxel level using small-volume corrections (Worsley et al., 1996). The dACC was defined as the dACC MRS voxel, the amygdala was anatomically defined using the Automated Anatomical Labeling atlas (Tzourio-Mazoyer et al., 2002), and the vmPFC (sphere with 10 mm radius centered at MNI_x,y,z_ = −2,52,−2) was defined similarly to the vmPFC previously implicated in studies of fear extinction and fear recall in healthy humans (see Milad et al., 2013).

## Results

### Psychophysiology

To assess the recovery of previously extinguished fear, we first assured successful conditioning to the CS+ on the physiological level. Skin conductance responses differed significantly between CS+ and CS− in the late phase of conditioning (*P* < 0.0001), thereby indicating successful acquisition of a fear memory to the conditioned stimulus (Table 1). During extinction training, participants showed a strong decrease in SCRs (late extinction vs. late conditioning for CS+ > CS−; *P* < 0.0001). During extinction retrieval, responses to the CS+ were significantly higher than to the CS− (*P* = 0.034). Skin conductance responses revealed a recovery of fear (i.e., the change in fear responses to the conditioned stimuli from the last two trials of extinction learning to the first response to the non-reinforced stimulus on the following day) to both the CS+ (*P* < 0.001) and the CS− (*P* < 0.001), suggesting that fear recovery generalized to both conditions across all participants. To assess individual differences in fear recovery that are specific to the CS+, an index of fear recovery was calculated for each participant (first trial extinction retrieval > last two trials of extinction for CS+ > CS−). Further supporting a recovery of fear, skin conductance responses to the CS+ were significantly higher during extinction retrieval than during habituation (*P* = 0.015), but not to the CS− (*P* = 0.204). To test our hypothesis of the involvement of GABA in the recovery of extinguished fear, a non-parametric correlation analysis showed that individuals with high GABA levels experienced stronger fear recovery (*r*_*s*_ = 0.28; *P* = 0.035; Figure 2C). Further exploration did not show an association between GABA levels and fear responses during the extinction or the retrieval phase separately (extinction: *r*_*s*_ = −0.18; *P* = 0.181; retrieval: *r*_*s*_ = 0.14; *P* = 0.311), suggesting that GABA mainly mediates the consolidation of the extinction memory from extinction training to extinction retrieval.

**Table 1:** **Skin conductance responses**.

**Fear learning phase**	**CS + (mean + *SD*)**	**CS − (mean + *SD*)**
Habituation	0.499 ± 0.312	0.499 ± 0.302
Early acquisition	0.844 ± 0.357	0.769 ± 0.336
Late acquisition	0.593 ± 0.304	0.282 ± 0.223
Early extinction	0.580 ± 0.334	0.497 ± 0.31
Late extinction	0.263 ± 0.304	0.179 ± 0.212
Early retrieval	0.521 ± 0.337	0.443 ± 0.317

*Means and standard deviations of the standardized skin conductance responses averaged over all participants for habituation, early (first two trials) and late phases (last two trials) of fear acquisition and fear extinction, and early extinction retrieval (one trial)*.

### Functional MRI

**Figure 3:**
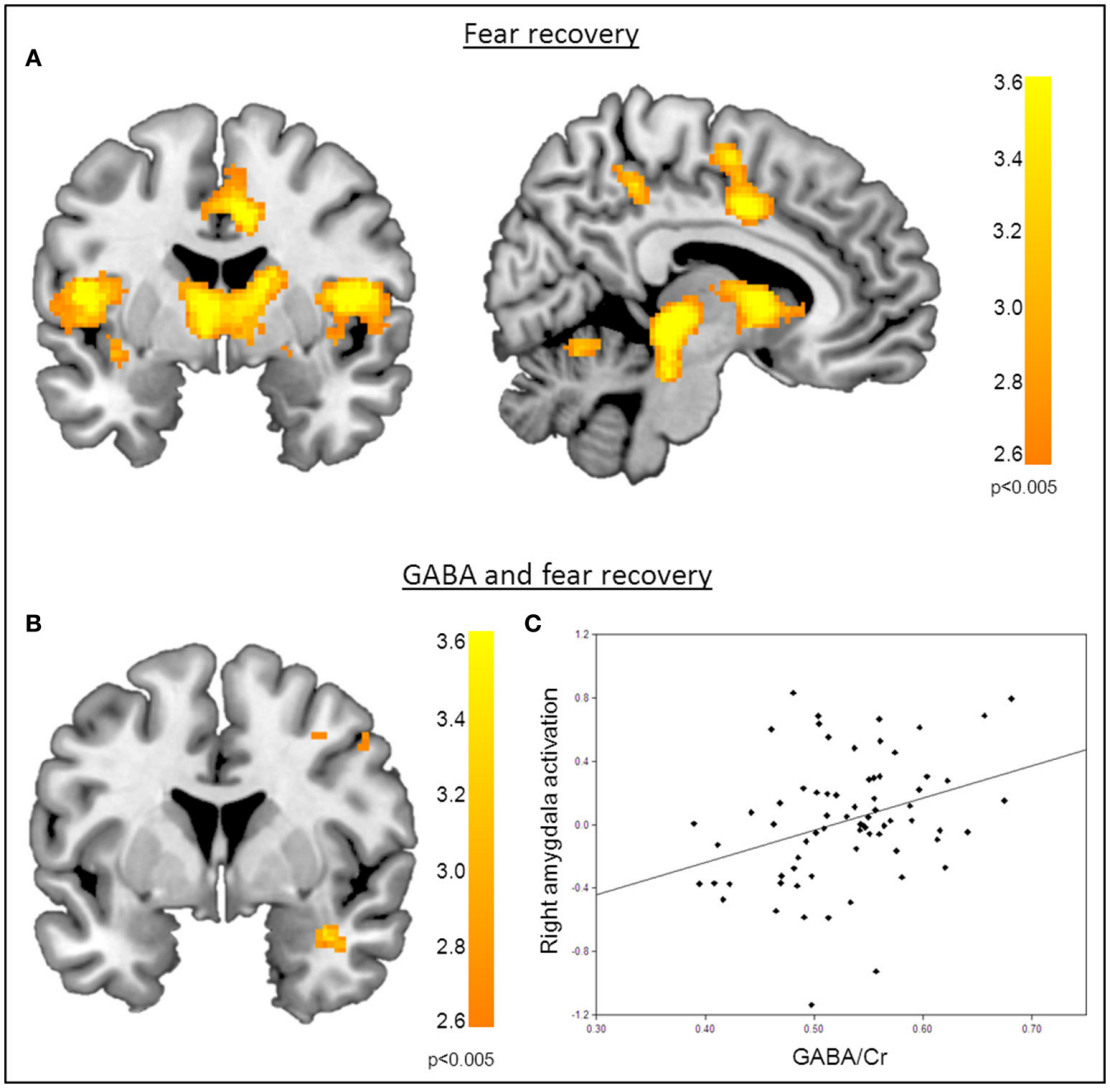
**Correlation of GABA levels and BOLD activity during fear recovery. (A)** Increased BOLD activity in the middle cingulate gyrus, the thalamus, the right putamen, and the supramarginal gyrus during fear recovery. **(B)** Voxel-wise correlation of GABA and right amygdala activation during fear recovery, showing that high GABA levels are associated with increased activation of the right amygdala (MNI_x,y,z_ = 36, 2, −24; P_SVC_ < 0.05). Threshold for displaying the images is set at *P* = 0.005, uncorrected. **(C)** Correlation between GABA concentrations and BOLD signal changes extracted from the right amygdala during fear recovery for illustration purposes, indicating that high GABA levels lead to increased fear recovery while low GABA levels are associated with a decrease in fear recovery.

**Figure 4:**
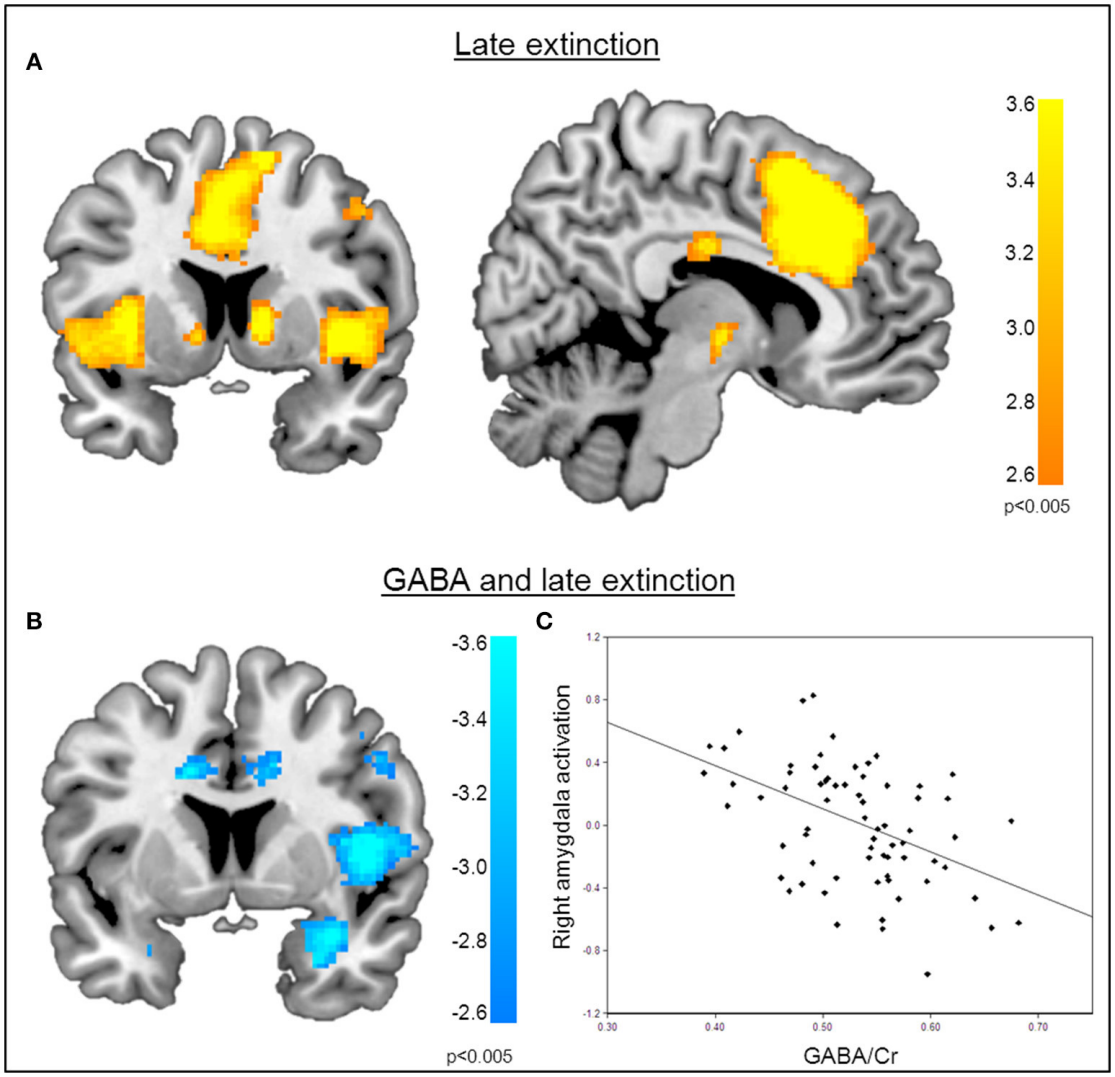
**Correlation of GABA levels and BOLD activity during late extinction. (A)** Increased BOLD activity in the dACC, the insula, and the superior medial gyrus for the “CS+ > CS−” contrast during the late phase of extinction. **(B)** GABA mediated deactivation of the right amygdala (MNI_x,y,z_ = 28, 4, −28; P_SVC_ < 0.05), right insula (MNI_x,y,z_ = 48, 8, 6; P_FWE_ = 0.008), and middle cingulate gyrus (MNI_x,y,z_ = 16, −22, 34; P_FWE_ < 0.001) during the late phase of extinction learning, indicating that high GABA levels are associated with diminished activation of the right amygdala. For displaying purpose, the image threshold is set to *p* = 0.005, uncorrected. **(C)** Correlation plot of GABA levels and activation extracted from the right amygdala during late extinction for illustration purposes, suggesting that high GABA levels facilitate extinction learning while low GABA levels hamper extinction learning.

Functional MRI data analysis confirmed the successful acquisition of fear. For all contrasts, activation to the CS+ in relation to the CS− is reported, and the results are corrected for multiple voxel-wise comparisons (*P* < 0.05; see methods). During the conditioning phase, activation in the fear related network including the middle cingulate gyrus, right insular lobe, right and left inferior frontal gyrus, right and left supramarginal gyri, left pallidum, left thalamus, right middle frontal gyrus, right and left precentral gyri, and the left amygdala was observed. To reveal the neural basis underlying the recovery of fear, we first assessed the brain regions involved in fear recovery. This analysis showed increased BOLD responses in regions within the fear network including the thalamus, the left post-central gyrus, the left inferior frontal and parietal gyrus, the right supramarginal gyrus, and the right rolandic perculum (Figure 3A, Table 2). To test our hypothesis of the involvement of GABA in the recovery of extinguished fear on the neural level, we included individual GABA concentrations in a voxel-wise correlation analysis of our fMRI data in order to investigate whether differences in activation in the fear network during fear recovery were mediated by individual differences in dACC GABA concentrations. This analysis showed that individuals with high levels of GABA showed a stronger recovery of previously extinguished fear in the right amygdala than participants with low dACC GABA levels (Figures 3B,C, Table 3). We subsequently analyzed the association between GABA and BOLD responses for the extinction learning and retrieval phases separately (Figure 4A). BOLD activity during the extinction retrieval phase was not significantly correlated with GABA levels. In contrast, we did observe a significant negative correlation between GABA concentrations and BOLD responses in the right amygdala, the cerebellum, the middle cingulate gyrus, and the right insula during the late phase of extinction learning (Figure 4, Table 3). No significant correlations were observed between SCR measures and BOLD activity during extinction, retrieval, and fear recovery.

**Table 2:** **Group activation for fear recovery, late extinction and early extinction retrieval**.

**Area of activation**	***P*-value FWE cluster-level**	**Cluster size # of voxels**	**T (peak)**	**Peak voxel MNI coordinates**
**FEAR RECOVERY: RETRIEVAL EARLY > EXTINCTION LATE**				
**CS+ > CS−**				
Thalamus	<0.0001	573	5.41	0, −28, −2
Left post-central gyrus	0.002	453	4.65	−48, −20, 24
Left inferior parietal lobule			3.52	−54, −28, 42
Left inferior frontal gyrus	0.026	247	4.54	−48, 6, 12
Right supramarginal gyrus	0.017	277	4.51	44, −30, 24
Right rolandic operculum			4.07	38, −34, 20
Right rolandic operculum	0.048	207	4.41	46, 2, 12
**EARLY RETRIEVAL**				
**CS+ > CS−**				
Right insula lobe	<0.0001	15034	8.62	34, 22, −4
Left insula lobe			8.36	−30, 24, −6
Left middle cingulate cortex			8.18	−46, 2, 310
Left middle cingulate cortex	<0.0001	7009	8.18	−4, 8, 38
Right middle cingulate cortex			7.90	6, 4, 36
Left SMA			6.64	−2, −2, 58
Right supramarginal gyrus	<0.0001	1513	6.47	56, −28, 28
Right supramarginal gyrus			6.31	64, −28, 30
Inferior parietal cortex			5.16	46, -36, 20
Right cerebellum	0.018	270	5.04	36, −48, −32
Right cerebellum			3.74	24, −54, −24
Right precentral gyrus	0.044	211	4.61	48, 4, 38
Right precentral gyrus			3.50	36, 0, 42
**CS−> CS+**				
Right paracentral lobule	0.017	274	6.42	8, −26, 62
Left paracentral lobule			5.80	−6, −32, 62
Left inferior temporal gyrus	0.001	456	5.94	−56, −6, −28
Left medial temporal pole			4.17	−48, 14, −34
Left middle temporal gyrus			5.09	−60, −14, −20
Right angular gyrus	<0.0001	630	5.91	52, −60, 26
Right angular gyrus			3.25	42, −64, 48
Left superior medial gyrus	<0.0001	1530	5.41	−8, 54, 28
Right superior frontal gyrus			4.80	22, 62, 10
Left superior medial gyrus			4.64	−10, 46, 44
Right precuneus	<0.0001	1355	5.35	6, −54, 26
Left middle cingulate cortex			4.53	−14, −48, 32
Right precuneus			4.42	12, −48, 12
Left precentral gyrus	0.025	247	4.83	56, −4, 28
Right post-central gyrus			3.69	42, −8, 28
Left angular gyrus	<0.0001	580	4.52	−52, −64, 24
Left angular gyrus			4.32	−44, −62, 26
Left angular gyrus			4.31	−42, −52, 24
Right post-central gyrus	0.005	354	4.47	36, −26, 48
Right precentral gyrus			4.08	34, −18, 60
Right precentral gyrus			3.72	32, −26, 58
Right superior frontal gyrus	<0.0001	694	4.37	20, 34, 52
**LATE EXTINCTION**				
**CS+ > CS−**				
Right insula lobe	<0.0001	1890	7.96	32, 24, −4
Left insula lobe	<0.0001	1193	6.13	−32, 22, −8
**LATE EXTINCTION**				
**CS+ > CS−**				
Left insula lobe			5.82	−30, 24, 2
Left superior temporal gyrus			3.65	−48, 6, −6
Right superior medial gyrus	<0.0001	2133	5.09	6, 26, 48
Right anterior cingulate cortex			4.73	4, 24, 28
Right supplementary motor area			4.66	6, 16, 52
Right supramarginal gyrus	0.019	292	4.37	60, −40, 30
**CS−> CS+**				
Right angular gyrus	<0.0001	5355	6.22	52, −64, 24
Left angular gyrus			6.16	−46, −72, 28
Left angular gyrus			5.71	46, −72, 32
Left superior frontal gyrus	<0.0001	1991	5.5	−18, 34, 46
Left superior medial gyrus			5.48	−10, 58, 14
Left superior frontal gyrus			5.14	−18, 42, 40
Right middle frontal gyrus	0.032	255	4.87	28, 24, 42
Right middle frontal gyrus			3.27	30, 22, 54
Right parahippocampal gyrus	0.003	448	4.82	28, −34, −8
Right parahippocampal gyrus			4.38	30, −30, −16
Right parahippocampal gyrus			4.11	34, −40, −8
Left hippocampus	0.003	302	4.72	−24, −20, −20
Left cerebellum			3.95	−20, −30, −26
Left hippocampus			3.63	−20, −8, −22
Right precentral gyrus	0.006	381	3.84	38, −24, 62
Right precentral gyrus			3.83	38, −22, 42
Right precentral gyrus			3.77	36, −20, 54

*All results are P <0.05 cluster-level FWE corrected with an initial height threshold of P = 0.001, uncorrected*.

^*^Small volume corrected with P_peak voxel_ < 0.05.

**Table 3:** **GABA and BOLD responses**.

**Voxel-wise correlations of dACC GABA and BOLD activation**				
**Area of activation**	***P*****-value FWE**	**Cluster size # of voxels**	**T (peak)**	**Peak voxel MNI coordinates**
**FEAR RECOVERY AND GABA**				
**Positive correlation**	Cluster level			
Fear network ROIs^*^	Peak level			
Right amygdala	0.022^*^	1	3.27	36, 2, −24
**LATE EXTINCTION AND GABA**				
**Negative correlation**	Cluster level			
Left cerebellum	<0.0001	719	5.07	−4, −40, −10
Right cerebellum			4.03	16, −42, −14
Cerebellum			3.84	6, −32, −18
Right insula lobe	0.008	291	4.04	48, 8, 6
Fear network ROIs^*^	Peak level			
Right amygdala	0.012^*^	7	3.46	28, 4, −28
**GABA mediated psychophysiological interactions using a right amygdala seed**				
**FEAR RECOVERY AND GABA**				
**Negative correlation**				
Fear network ROIs^*^	Peak level			
vmPFC	0.023^*^	47	3.51	10, 52, −2
**LATE EXTINCTION AND GABA**				
**Positive correlation**				
Fear network ROIs^*^	Peak level			
vmPFC	0.003^*^	219	4.21	0, 44, −2

*All results are P <0.05 cluster-level FWE corrected with an initial height threshold of P = 0.001, uncorrected*.

* *Small volume corrected with P_peak voxel_ <0.05*.

### Functional connectivity

**Figure 5:**
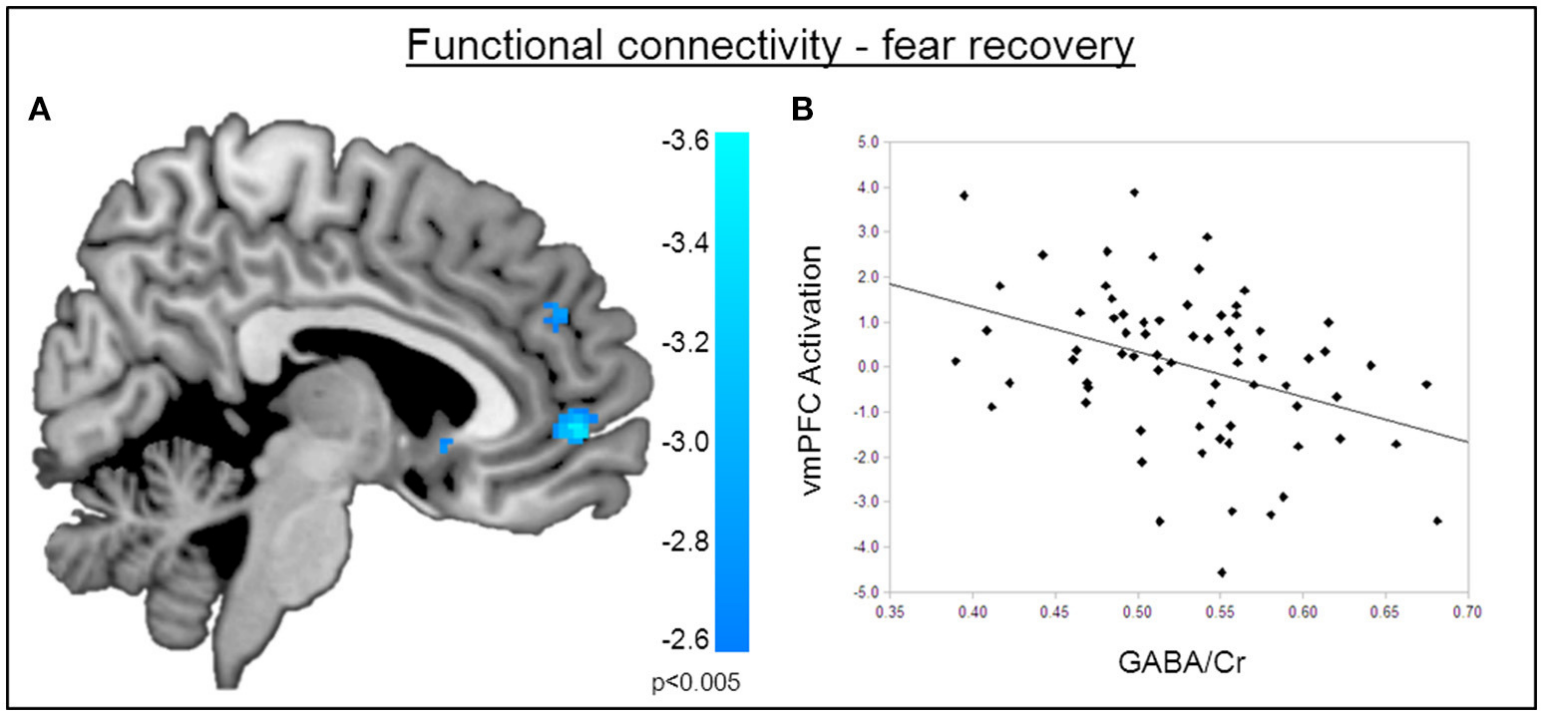
**Functional connectivity during the recovery of fear. (A)** Psychophysiological interactions with the right amygdala seed region during fear recovery revealed a negative voxel-wise correlation of GABA mediated amygdala-vmPFC connectivity during fear recovery (MNI_x,y,z_ = 2, 52, −2; P_SVC_ < 0.05). Threshold for displaying the images is set at *P* = 0.005, uncorrected. **(B)** Correlation plot of dACC GABA levels and deactivation of the vmPFC showing that high GABA levels are correlated with diminished amygdala-vmPFC connectivity compared to low GABA levels.

**Figure 6:**
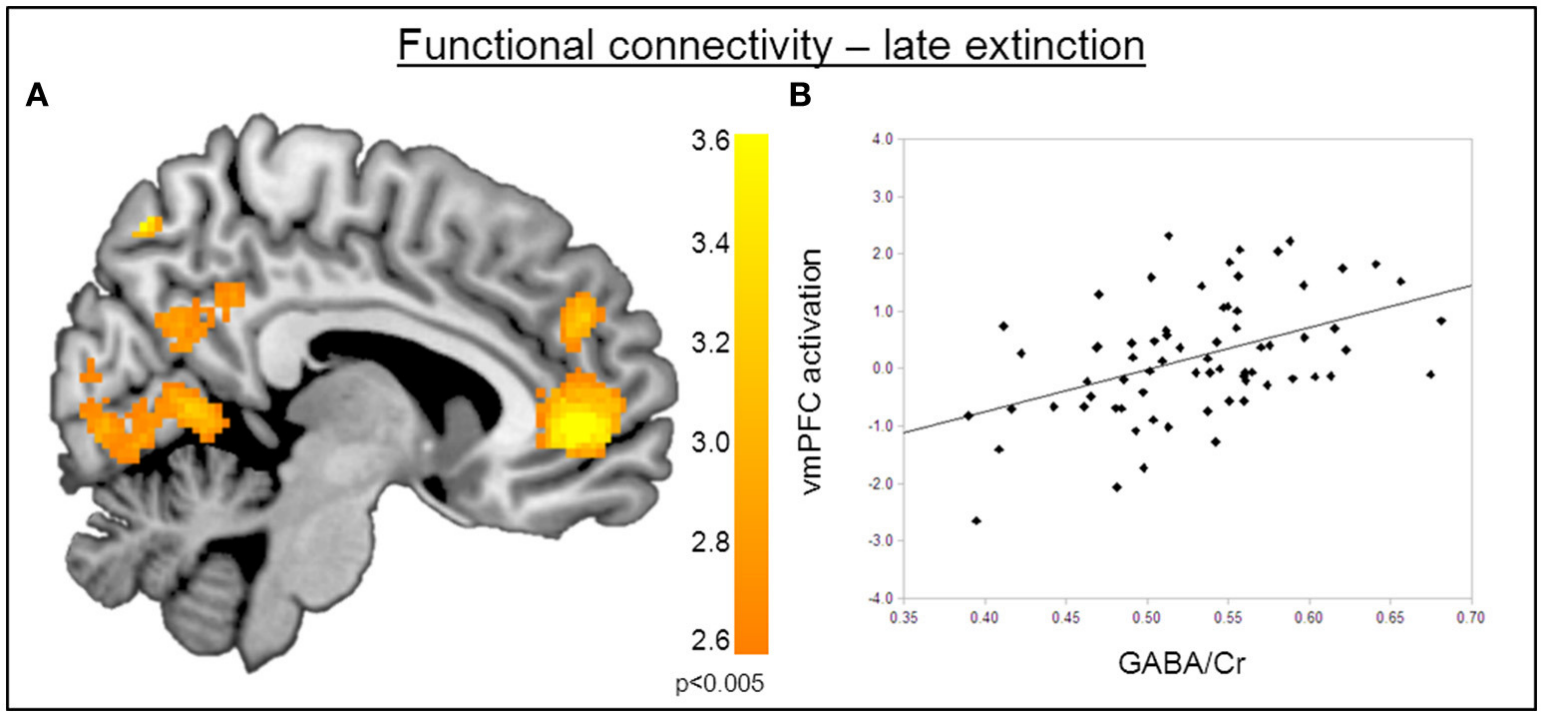
**Functional connectivity during late extinction. (A)** A psychophysiological interaction analysis revealed that high levels of GABA mediate an increase in connectivity between the amygdala seed region and the vmPFC (MNI_x,y,z_ = −4, −40, −10) during extinction (P_FWE_ < 0.05). **(B)** Correlation plot illustrating a positive relation between dACC GABA levels and amygdala-vmPFC connectivity.

The functional MRI results reported above showed a positive correlation between GABA concentrations in the dACC and activity in the right amygdala during the recovery of fear. To explain the functional interplay between these two regions, we aimed to investigate functional connectivity in order to examine whether and to what extent this connectivity may be mediated by dACC GABA concentrations. As GABA concentrations in the dorsal ACC correlated with BOLD signal in the right amygdala, we further explored potential interrelations between these core regions and the vmPFC as a modulatory inter-connected region within this circuit. A psychophysiological interaction analysis revealed a negative correlation of dACC GABA and functional connectivity between the vmPFC and the right amygdala during fear recovery (Retrieval_(*CS*+ > *CS*−)_ > Extinction_(*CS* > *CS*−)_). Reduced connectivity to the CS+ (relative to the CS-) during extinction learning compared to extinction retrieval suggests that high dACC GABA levels may lead to reduced inhibitory control of the vmPFC on amygdala function, and thereby result in increased fear recovery (Figure 5, Table 3). To disentangle contributions of the associated phases, we separately examined connectivity for the early retrieval and the late extinction phases. In contrast to the negative GABAergic impact during fear recovery, the results showed a positive association between dACC GABA on amygdala-vmPFC coupling separately during late extinction during CS+ trials relative to CS− (Figure 6, Table 3). During the retrieval phase, GABA did not mediate connectivity of the amygdala and any other region.

## Discussion

This study reveals that the main inhibitory neurotransmitter in the human brain is involved in the regulation of the recovery of fear. Providing insight into the neurochemical basis of fear learning, these findings highlight the opposing role of dACC GABA in the successful extinction of learned fear and its retention over time. Results show that individual differences in dACC GABA predict the propensity for the recovery of fear the following day on both the physiological and the neural level within an interconnected brain circuit in a large sample of healthy individuals. GABA concentrations appear to influence the recovery of fear through modulation of amygdala responsivity, indicating that high baseline GABA levels are associated to increased fear recovery as suggested by both skin conductance responses and BOLD data. Separate analyses of extinction training and the extinction retrieval showed that GABA diminished fear responses to the conditioned stimulus in a large fear network during the late phase of extinction but not during extinction retrieval. No significant association between GABA and physiological responses was observed during these distinct phases, suggesting that GABA primarily affected our index of fear recovery that assesses the increase in skin conductance responses from late extinction learning to early extinction retrieval. Although the physiological results suggest that dACC GABA has no influence on extinction learning or extinction retrieval separately, the neuroimaging results during the extinction training session also suggest that dACC GABA is involved in the inhibition of conditioned fear responses during extinction learning. Together with the fear recovery results, this suggests that dACC GABA is differentially associated with the extinction of fear and its later recovery, and may therefore be particularly important in the consolidation of extinction memory. While our results and study design do not provide any information on causal effects of GABA on functional variability, this interpretation is consistent with findings from rodent studies showing that increasing inhibitory neurotransmission after extinction training interferes with the consolidation of extinction (McGaugh et al., 1990; Akirav, 2007). While the disrupting role of GABA in the consolidation of extinction is well accepted, findings are less consistent for extinction learning. Several studies suggest that increased GABAergic neurotransmission facilitates extinction learning, whereas others have shown no influence of GABA on extinction learning (Harris and Westbrook, 1998; Akirav et al., 2006; Hart et al., 2009; Makkar et al., 2010). However, despite the absence of GABA and psychophysiological correlations, our fMRI findings indicate support for the former, and suggest that GABA has a facilitating role in human extinction learning.

These opposing effects have been observed for both systemic and local interventions. Local interventions mainly focused on three brain regions: the infralimbic cortex, the prelimbic cortex, and the (basolateral) amygdala. Interference of GABAergic neurotransmission in the infralimbic cortex and amygdala have been shown to disrupt extinction consolidation, but the prelimbic cortex has not been found to play a role in extinction consolidation in rodents (Akirav, 2007; Laurent and Westbrook, 2009). The prelimbic cortex is assumed to be homologous to the human dACC, but in contrast to rodent studies, our findings do show an inhibitory role of the human dACC in extinction learning and consolidation. A possible interpretation of the opposing association between GABA and the consolidation of extinction memories could be that dACC GABA levels interfere with neuronal activity in the amygdala which would be required to successfully consolidate and express the newly generated memory association (Akirav et al., 2006; Vidal-Gonzalez et al., 2006; Likhtik et al., 2008). This could happen either directly through dACC-amygdala connectivity or through interconnected brain regions of the dACC and amygdala which may be modulated by dACC GABA levels. One possibility suggested by our results is that this association is mediated by the vmPFC. These findings are in line with previous findings that showed decreased amygdala-vmPFC coupling during memory consolidation after a fear conditioning paradigm (Feng et al., 2014). Functional connectivity data revealed GABA modulated coupling of the amygdala and the vmPFC during the recovery of fear, with high levels of dACC GABA predicting reduced connectivity between these regions, potentially resulting in diminished inhibitory control of the vmPFC on the amygdala. A lack of modulatory control on the amygdala could lead to an increase in amygdala activity, thereby promoting the recovery of fear responses. These findings extend previous evidence by Delli Pizzi et al. (2016b) who found mPFC GABA levels to be inversely related with negative functional coupling of the amygdala and the vmPFC during resting fMRI. In addition, they found trait anxiety to be inversely related to functional amygdala-vmPFC coupling. Similarly to BOLD activity results, the opposite effect of GABAergic abundance on functional connectivity between the amygdala and the vmPFC was observed during fear extinction. Enhanced coupling of the amygdala and the vmPFC was shown to be mediated by high levels of GABA, suggesting that this increase in connectivity facilitates prefrontal control of the amygdala, thereby facilitating the extinction of conditioned fear.

In the light of interpreting our findings, it is important to highlight that the main aim of this study is to investigate factors that contribute to individual differences in human fear recovery, rather than providing a mechanistic account of how GABA regulates fear learning as it is beyond the scope of this study and the available techniques to investigate the fine-grained fear circuitry identified in animal studies. With regards to our GABA measurements, it is important to point to the lack of clarity with regards to the interpretation of the GABA MRS signal source and how it relates to synaptic activity (Stagg et al., 2011; Rae, 2014). Potential sources can be cytoplasmic, vesicular, and free extracellular GABA with metabolic, neurotransmission, and neuromodulatory functions, respectively. Both vesicular and free extracellular concentrations have been suggested to be represented in the GABA MRS signal. However, a clear distinction between GABA pools is not possible and remains subject to discussion (Martin and Rimvall, 1993; Belelli et al., 2009). Furthermore, the baseline measurement of GABA levels on the first experimental day and the interpretation of our results assume the stability of GABA levels in the dorsal ACC over longer periods of time. Although prefrontal GABA levels have been shown to drop during acute stress (Hasler et al., 2010), resting GABA measurements have shown good reproducibility for within-day measurements as well as for an interval of 7 months (Evans et al., 2010; Near et al., 2014). Individual differences in GABA levels should therefore remain stable over the 24 h time period between sessions. Even though the aim of this study was to examine correlations of baseline GABA levels and fear recovery, investigating changes in GABA levels throughout the experiment would provide further important insights into role of GABA on fear responses. Future studies should aim at assessing GABA levels at different time points throughout the fear learning paradigm and how this relates to baseline GABA concentrations. Another potential limitation of the study is the high number of excluded spectra. For specificity of voxel location and voxel dimensions, we aimed for precise coverage of the region of expected dACC BOLD activity during fear conditioning. The resulting voxel volume of 16 cm^3^ is relatively small for GABA-edited MRS and resulted in a substantial number of spectra that were of insufficient quality.

These results provide a first step to understand how differences in brain GABA concentrations may relate to fear related disorders. Our results show that healthy individuals with high GABA levels show higher fear recovery. A recent study in patients with PTSD showed that ACC GABA concentrations are significantly higher in patients compared to healthy controls (Michels et al., 2014). In addition, the administration of a GABA agonist acutely reduces anxiety but also reduce the efficacy of exposure therapy in PTSD (Rothbaum et al., 2014). Both studies point to increased GABA concentrations and GABAergic neurotransmission as a critical aspect in PTSD. Our results suggest that increased GABAergic activity reduces the consolidation of extinction training and leads to more fear recovery, which may explain the high levels of fear in PTSD patients and adverse effects of GABA agonists on long-term outcome.

In conclusion, these results show that individual differences in dACC GABA levels are associated with differences in the intensity of fear recovery in healthy young men. We suggest that this might potentially be through differential modulation of activity and connectivity within a tightly interconnected network involving the amygdala, dACC, and vmPFC. Our findings particularly highlight that correlations between GABA and both neural and psychophysiological fear measures differ not only depending on neurotransmitter quantity but are also dependent on the phase of fear learning, partially revealing opposing effects during fear recovery and extinction learning. While high GABA concentrations appear to benefit extinction learning, associations with the consolidation of extinction point into a negative direction. Our findings are intriguing as they link findings from rodents to humans which should be further extended to anxiety related disorders.

## Author contributions

NL and GAvW designed research; NL and JvL performed research; NL analyzed data; NL and GAvW wrote the first draft; JvL, NP, DD contributed to and approved the manuscript.

## Funding

This work was supported by the Netherlands Organization for Scientific Research (NWO/ZonMW 916.11.037) and by the AMC Reseach Council (110913).

### Conflict of interest statement

DD is a member of the advisory board of Lundbeck. He receives occasional fees from Medtronic for educational purposes. The reviewer MT and handling Editor declared their shared affiliation, and the handling Editor states that the process nevertheless met the standards of a fair and objective review. The other authors declare that the research was conducted in the absence of any commercial or financial relationships that could be construed as a potential conflict of interest.
