# Supplementary material for: GABA Concentrations in the Anterior Cingulate Cortex Are Associated with Fear Network Function and Fear Recovery in Humans
Source: Front Hum Neurosci. 2017 Apr 27;11:202. doi: 10.3389/fnhum.2017.00202 (PMC5406467; doi:10.3389/fnhum.2017.00202)
Supplement: Supplementary file 1 [file DataSheet1.DOC]

**Supplemental Information**

*Material & Methods*

Participants

Before entering the study, participants were screened to have normal or corrected-to-normal vision, no average use of more than three alcoholic beverages per day, no weekly use of recreational drugs, no habitual smoking, and no current or history of psychiatric, neurological, or endocrine disease, and recent irregular sleep/wake rhythms. Women were not included due to menstrual cycle influences on both GABA levels and fear learning (De Bondt et al, 2015; Epperson et al, 2005; Lebron-Milad and Milad, 2012; Milad et al, 2006). Participants were instructed to not smoke 24 hours prior to the first experimental session until the end of the experiment. Recreational drug usage was prohibited starting 72 hours before entering the study until the end of the experiment.

Procedures

MR compatible carbon electrodes (Kendall H135 TSG) were placed approximately 4 cm apart and were attached to a Digitimer DS7A with a source voltage of 400V and pulse duration of 2000µs. Two Ag/AgCl electrodes were attached to the phalanges of the index and middle finger of the left hand. An MRI compatible EGI (Electrical Geodesics, Inc., Oregon, United States) GSR setup was used to enable SCR recordings at a sampling rate of 250Hz using NetStation software (version 4.5.2).

MRI data acquisition

The MRI protocol included a MPRAGE anatomical scan to plan the dACC MRS voxel (TR/TE=8.2ms/3.8ms, FOV=240x188mm², voxel size=1x1x1mm³, 220 slices). Whole brain echo planar images (EPI) were acquired with the following parameters: TR/TE=2000ms/28ms, slice gap=0.3mm, FOV=240x240mm², voxel size=3x3x3mm³, and 37 slices.

MRS

Selective inversion was achieved using 15.64 ms sinc-center pulses (64Hz bandwidth) at the 1.91ppm resonance of GABA on all odd numbered acquisitions. On even-numbered acquisitions, selective inversion pulses were applied symmetrically around the water resonance. Difference spectra were obtained using in-house software.
